# Interaction and self-correction

**DOI:** 10.3389/fpsyg.2014.00798

**Published:** 2014-07-23

**Authors:** Glenda L. Satne

**Affiliations:** Center for Subjectivity Research, University of Copenhagen, CopenhagenDenmark

**Keywords:** interaction, self-correction, naturalism, normativity, evolution, conceptual abilities

## Abstract

In this paper, I address the question of how to account for the normative dimension involved in conceptual competence in a naturalistic framework. First, I present what I call the naturalist challenge (NC), referring to both the phylogenetic and ontogenetic dimensions of conceptual possession and acquisition. I then criticize two models that have been dominant in thinking about conceptual competence, the interpretationist and the causalist models. Both fail to meet NC, by failing to account for the abilities involved in conceptual self-correction. I then offer an alternative account of self-correction that I develop with the help of the interactionist theory of mutual understanding arising from recent developments in phenomenology and developmental psychology.

## INTRODUCTION

Conceptuality traditionally seems to impose specific challenges to the possibility of a naturalistic account of mind. The issue I address in this paper is how to specify the normative abilities that are associated with conceptual competence in order to meet a very popular challenge in recent developments of philosophy of Mind, what I call the naturalist challenge (NC). I do not intend to provide a complete or even general account of conceptuality but, more modestly, I try to specify certain conditions that a naturalistic account of conceptuality should accommodate, conditions that define a framework of specific questions and concerns, in particular in relation of our capacities of conceptual self-correction, that lead us, I argue, to prioritize a certain approach *vis-à-vis* others: the interaction theory of mutual understanding. In the context of that general approach, I claim it is possible to account for self-correction in a way that is compatible with the challenge at issue.

Addressing the problem of conceptual competence within a naturalist framework makes it necessary to meet the NC, that is, to account for:

(1) the evolutionary path from creatures without language or thought to creatures with both abilities without postulating any explanatory and/or evolutionary gap^1^.
(2) the capabilities of learning or acquiring conceptual contents – and a natural language – without producing or presupposing any explanatory and/or evolutionary gap or committing to the existence of non-natural entities.

And a further constraint:

(3) Answers to (1) and (2) must be able to justify the attributions of intentional attitudes to children and non-human animals^2^.

There are two main strategies that have been adopted toward this challenge. Both of them, when broadly construed, define two general models of conceptual abilities that may be described in terms of the adoption of a first-personal perspective or a third-personal one. The first one, that can be called the first-personal model, includes those attempts to understand conceptual abilities that focus on the individual’s brain states, conceiving them as dispositions or informational states that are related in appropriate ways to the environment such that they can be conceived as constitutive of the competence involving a specific concept. According to this model, NC is met because the explanatory work is made by a naturalistic specifiable notion, i.e., one that can be found pervasively in the natural sciences, the notion of *causation*. What makes a state constitutive of the competence according to a concept is its being properly *caused* by that to which the concept refers to or is about. In this sense, these approaches are causalist accounts of the nature of conceptual competence.

The second approach I examine focuses not on the individual brain states but on the attributive standpoint of an interpreter that can understand an individual’s behavior conceptually, thus undertaking a third-personal perspective. This strategy is known as an interpretationist account of conceptual abilities. NC is met – so the defenders of this position claim – because this perspective is not committed to there being any specific reality of concepts over and above the interpretational activity of taking the behavior at issue to be explained in terms of the attribution of the concepts in question.

My aim in this paper is twofold:

(a) To argue that both causalist and interpretationist accounts of conceptual abilities are unable to meet NC. The reason for this failure is that both models are inadequate to account for mistakes in the application of concepts.
(b) To offer an alternative model – a second-personal interactionist model – that meets NC by accounting in a different way for the ability to make conceptual mistakes.

## CONCEPTUAL ABILITIES: BASIC NOTIONS AND CONSTRAINTS

There seem to be good reasons to think that no matter how we define conceptual abilities nor the position we assume concerning the scope of conceptual content and its articulation with experience, being able to apply concepts presupposes as a necessary condition – though of course not sufficient –being able to distinguish between correct and incorrect applications of them in actual cases. This is what we may call the *normative constraint on conceptual abilities*^3^.

Such constraint can be defined as follows:

(1) To have conceptual abilities involves as a basic ability being able to correctly apply concepts, i.e., to distinguish between correct and incorrect uses of them in given circumstances. This means that in order to account for the nature of conceptual abilities, it is necessary to account for the ability to recognize a correct application of a concept and distinguish it from incorrect ones.
(2) Conceptual competence does not only involve recognizing an incorrect use of a concept but also implies to be willing, in that case, to abandon that use and modify it if necessary, i.e., to self-correct when noticing an incorrect use of a concept by oneself. This means that in order to account for the nature of conceptual abilities, it is necessary to account for self-correction.

Further precisions are required in order to understand correctly the constraint. As it may be apparent, the normative dimension involved in the ability to apply concepts involves the possibility of error.

There are nevertheless two notions of error or mistake that must be distinguished. In particular, there are two different kinds of mistakes that we attribute to others in their use of concepts. On the one hand, we may attribute error to someone when she misapplies a concept. I call this *misapplication or conceptual mistake*. On the other, we may attribute lack of competence to a person regarding a concept when she lacks the concept or is simply not applying the concept at all. This I what I call *absence of application*. Such distinction will prove especially fruitful when assessing whether a model of conceptual abilities can fulfill the normative constraint accommodating the requirements of NC.

Consider the following cases:

(i) John has been adding correctly and suddenly says “57 + 124 = 171.”
(ii) John does not know how to reply to a question regarding the sum of two numbers (he answers randomly, or he simply shrugs his shoulders).

In the first case, we attribute to John that he is adding wrongfully, in the second one that he simply is not adding. While (i) is a case of conceptual mistake, (ii) is just a case of absence of application. The crucial difference lies in the fact that while in the former case the concept in question is relevant to the evaluation of the action, i.e., is relevant for the way in which the performance is carried out; in the second case the concept is not relevant for explaining his performance, it is simply absent^4^. Following our previous constraints, to account for the normative constraint specified in (1) and (2) above, it is necessary to be able to account for the abilities that underlie the attribution to a subject that she is committing a mistake in the use of a concept (conceptual mistake), and to distinguish that case from a case in which the subject is simply not applying the concept (absence of application), i.e., it is necessary to account for when and how someone who uses a concept, commits and recognizes conceptual mistakes and accordingly self corrects her use and to distinguish that case from one in which the subject is not applying the concept at all.

How should we then understand self-correction in application of concepts? Self-correction in the relevant sense seems to involve three dimensions of performance:

(a) The application of concepts (the actions of applying or misapplying a concept).
(b) The ability to evaluate (a).
(c) The modification of (a) according to the results of (b).

As it will be shown in the following sections, both causalist and interpretationist accounts of conceptual abilities fail when accounting for the distinction between cases of misapplication or conceptual mistakes and cases of absence of application and the consequence of this failure is their inability to meet NC.

## THE CAUSALIST CONCEPTION OF CONCEPTUAL ABILITIES^5^

The way in which competence regarding a specific concept X can be defined in causal terms is the following:

John is competent with respect to concept X iff given certain conditions C, John is disposed to apply X to y iff X(y) is true^6^.

In this framework, conceptual mistakes are modeled in terms of the failure of a mechanism: conditions C are not given. The reason for this failure might be internal to the mechanism, that is, that the mechanism is malfunctioning or it might be the absence of one of the enabling conditions required for the mechanism to work.

I claim that when assuming such way of understanding conceptual competence, there is no non-question-begging way of distinguishing between conceptual mistakes and absence of application.

It is important to bear in mind that if John’s mistakes can be accounted for equally as conceptual mistakes according to a concept, or as a case of lack of application, there would be no way to account for the capacity to make conceptual mistakes. Say John says “5 + 6 = 12.” We would be immediately inclined to think he was adding and adding wrong. But he could equally be performing a different operation, say, +^∗^, and doing it correctly. If an account of conceptual capacities could not distinguish between both cases, it would fail to explain what is for John to have any conceptual ability and to distinguish this from the case where this ability is merely absent.

The causalist model fails to provide a plausible distinction between conceptual mistakes and absence of application at least for two reasons:

The first reason is that, according to this model, the subject’s reactions/dispositions to apply concepts can be described in terms of different concepts. So in this model it is not possible to distinguish between cases of conceptual mistakes and cases of lack of application. As Boghossian (1989)^7^ famously pointed out, the same reactions can be described using different concepts. This further requires for the model to distinguish different responses as appropriate or not in specific contexts, and in order to identify the proper set of responses we need to distinguish the good cases from the bad ones, conceiving these as cases in which conditions C fail, in the example at issue conditions C would include John cognitive mechanisms working fine, including normal functioning of attention, memory, etc. The problem is that we can only distinguish the two cases by using the concept we want to reconstruct, stipulating which is the concept in question, for example, stipulating that when John says that “6 + 5 = 12,” he is using the concept of addition. But this means that we have to presuppose its content without accounting for it in terms of reactions, opening an explanatory gap. Importantly, there is no distinction between absence of application and misapplication that does not depend on stipulating the concept at issue and thus presupposing the pertinence of that very distinction. It is important to bear in mind that this problem rises independently of whether the account takes these processes to occur at the subpersonal level or at the personal one. In either case, there is no non-question-begging way of distinguishing that the behavior accords with one concept and thus is a case of conceptual mistake and not mere absence of application of that concept^8^. Thus, the proposal fails to meet NC^9^.

The second reason why this view fails to make the distinction between misapplication and absence of application is that this account does not give a proper account of self-correction. According to this kind of theory, the source of error is a failure in conditions C, but this kind of error is independent of the subjects being able to identify it in practice. The mistakes are of such a nature that the subject may be unable to identify them (direct access to them could even be impossible for the subject) and modify his use of concepts according to the identification of error and its sources.

In fact, conditions C are not conceptually linked to the concepts the subject is applying or trying to learn. But self-correction seems to be a key ability to account for the process of learning new conceptual contents through training. Can this theory account for the connection between the identification of mistakes and conceptual abilities that seem constitutive of the process of learning conceptual contents and linguistic terms associated with them? As shown before, they cannot. This amounts to a failure to meet NC, since there is an explanatory/evolutionary gap concerning how new concepts are learnt and from this perspective the fact that concept users are able to apply concepts correctly and self-correct themselves if mistaken seems to be a complete mystery.

However, someone may hold that there are second order dispositions to evaluate reactions (corresponding to the component (b) of self-correction described above). The idea would then be that by positing them it is possible to account for self-correction and still defend a purely dispositional account of conceptual competence^10^.

But a similar problem arises: if those (second-order) dispositions were fallible and learnt, they would require dispositions of higher order to be learnt. This involves a vicious regress. If, on the contrary, those dispositions are not fallible and learnt, they are some kind of *sui generis* dispositions. This leaves their nature unexplained: are they to be conceived in causal terms? It seems that they must not be, in order to avoid the previous difficulties, but then another notion of conceptual ability must do the work here. This leads to an explanatory gap. Thus, the theory fails to account for NC (2) since it cannot explain the learning and acquiring of conceptual contents in a naturalist way (it fails by opening an explanatory gap when introducing the *sui generis* dispositions involved in self-correction). And it also fails to account for NC (1) since its inability to account for self-correction shows a corresponding failure to draw crucial distinctions between the capabilities of artifacts and other sorts of entities, some of them capable of self-correcting in ways that others are not. There is, according to this model, only one basic kind of mechanism that explains all of these *prima facie* different phenomena. But then the proposal fails in explaining the nature and complexity of different abilities in terms of more basic or previous ones, and so fails in drawing the relevant distinctions between abilities and capabilities of different complexity in a natural and gradual scale^11^.

## THE INTERPRETATIONIST ACCOUNT OF CONCEPTUAL ABILITIES^12^

I have presented three dimensions that are involved in self-correction:

(a) The application of concepts (the actions of applying or misapplying a concept).
(b) The ability to evaluate (a).
(c) The modification of (a) according to the results of (b).

If causalism thinks of level (b) by analogy with (a) and fails to account for (c), interpretationism stresses level (b).

Briefly sketched, according to this model to be a conceptual creature is to be a language user. Both notions are accounted for in terms of interpretation: to be a conceptual creature is to be able to interpret other creatures’ actions as meaningful. The interpretation of language is just a part of the global task of attributing meaning to other creatures’ behavior. To interpret someone is to attribute meaning to their conduct conceiving it as oriented by wishes and beliefs in the context of a common perceived world. In sum, to interpret someone is to implicitly construct a theory about the content of their beliefs, wishes and the like, in the context of a world where both the interpreter and the interpretee are commonly situated.

The emphasis in this view lies then on component (b), the evaluation of the actions of a subject according to concepts. Accordingly, the model defines conceptual competence as follows:

John is competent with respect to a concept X iff John applies X to y only when the interpreter would apply X to y, or y is such that the interpreter would have applied X to it, had his beliefs been slightly different in a way that matches John’s (assuming that the attribution of the belief that y is X to John respects principles of rationality, charity, humanity and causality regarding the interpretation of John’s behavior as a whole)^13^.

The attribution of error – in the sense of conceptual mistakes – is captured as a difference between the perspective of the interpreter and the perspective of the interpretee regarding a special case of application. This may happen in a number of ways. It might be the case that the subject makes a perceptual judgment about something that is openly accessible to both the interpreter and the speaker or it might be that the claim involves a judgment that is not immediately connected to the commonly available perceptual evidence for both speaker and interpreter. Both cases are structurally similar according to this theory, even if they are distinct in terms of the role that each kind of judgment plays for the interpreter to construct the ongoing understanding of the speaker’s discourse. While the former constitutes the beginning of the interpretational process, the latter depends on previous judgments concerning what the speaker is taken to believe, intend and desire.

The structural similarity resides in that, for the interpreter, to be able to interpret the speaker’s judgment she would have to assume that the speaker shares with her a vast optimized majority of true beliefs. Because of the general theory about what the speaker is trying to convey at that particular moment, the interpreter can then attribute local mistakes to what is asserted. The difference between the two cases is then that in order for the interpreter to make sense of what is being asserted she would start by attributing to the speaker that he is related to the same environment that she is and by that token that he perceives and holds to be true beliefs about that environment that are the same as those she herself holds. It is only with specific evidence to the contrary that the interpreter will withdraw this particular attribution and then attribute to the speaker an error of judgment regarding what both are commonly perceiving. Error will then be explained as a matter of difference between what the interpreter takes to be the case and what she can make sense of the speaker trying to convey, taking into account all the other evidence she has about his beliefs, desires, and the like. The cost of attributing error to commonly held judgments is so vast that rationality constraints on the interpretation dictate to attribute a difference between her perspective and the one of the speaker regarding some other judgment. This is all left on the hands of the interpreter who can then make sense of the behavior in different ways, all compatible with the evidence. The rule is always to attribute the less possible mistake, which is just the content of the principle of charity that governs interpretation.

This model turns out to be problematic when trying to distinguish between conceptual mistakes and absence of application – and hence to account for conceptual abilities. There are at least three difficulties worth mentioning:

(1) Following the principles of interpretation, the conduct of the interpretee can be described either way, as a case of misapplication of a particular concept or as a case of absence of application. The concept of error is just a tool for interpreting another person’s behavior, an attribution that can be canceled by a better interpretation. Hence, this theoretical reconstruction does not distinguish between conceptual mistakes and absence of application.
(2) The theory presupposes the notion of error precisely as a notion that the interpreter can – and has to – use. To be an interpreter is to have the concept of belief: to be able to interact with somebody else is to be able to attribute beliefs to him. The concept of belief in turns presupposes having the concept of error, of falsehood. But the theory does not explain how this concept is gained but rather presupposes the need of such a tool; and thus produces an explanatory gap in accounting for the mastery of conceptual abilities. Moreover, the acquisition of thought, i.e., of the concept of belief, is conceived as emerging from an evolutionary gap, since the model seems to be committed to the idea that at some point this ability emerges but is not clear how it develops from previous more basic ones. The model then fails to meet both NC (1) and NC (2).
(3) Because of the identification between thought, talk and interpretation, the theory cannot account for the ability to entertain thoughts but not to speak a language (as may be the case with some non-human animals), or for the possibility to have rudimentary forms of thought and talk (as in the case of young children), and *a fortiori* cannot describe those abilities as forming a continuous path of little steps.

In sum, the model fails to meet NC (2), since it cannot explain the learning of conceptual abilities as a gradual process. This implies an explanatory gap regarding the acquisition of language, in particular in the acquisition of the concept of error to be attributed to oneself and others. For these reasons, the model cannot account either for continuity in nature, i.e., for the way in which complex abilities of some natural entities emerge through gradual changes and combinations of more basic capabilities exhibited by other natural entities, and this is a failure to meet NC (2). And this also means that this kind of theory cannot explain our attribution of thought to animals and children, such attributions would be at the most mere “ways of talking^14^,” that would not be justified in terms of the abilities exhibited by the behavior of such agents, i.e., the theory cannot answer to NC (3). This leaves unexplained the nature of their capacities and the connection between their ways in the world and ours.

## MY STRATEGY TO MEET NC: CONCEPTUAL MISTAKE AND STANDARDS OF CORRECTION

The above considerations have shown that both causalist and interpretationist accounts fail when accounting for component (b) of self-correction, i.e., the ability to evaluate the performance (a). Thus, in order to overcome their difficulties we need to offer an explanation of level (b) of the self-correction dimensions that (i) is not reduced to mere causal reactions, as in the case of causalist models. The strategy is to include an evaluative component that is not conceived in terms of level (a). Second, the account of (b), must (ii) not presuppose articulated contentful thought, as is the case of interpretationists account. As in the previous cases, the account of (b) needs to (iii) have the relevant consequences for (c).

Before presenting my strategy, there are some distinctions and precisions that are worth making. The aim to give an account of conceptual competence seems to be a highly ambitious one and there are of course a number of different proposals all of which would deserve to be seriously taken into account when analyzing what the correct answer to NC might be. One issue that is of particular relevance in this domain is the distinction between conceptual and non-conceptual content. As it is known, many current theories of conceptual competence attempt to address what I am calling the NC precisely by drawing that distinction. Nevertheless, I neither address this specific topic in this paper nor I explore alternative attempts to bridge the gap between the conceptual and non-conceptual domains^15^. I can dispense of doing that since what I would be arguing for is neutral to those further worries. It should be noted that my claim is not that all cognition should be conceptual but rather that to account for conceptual abilities while meeting NC, the account needs to meet the normativity constraint. So my point is the following: no matter where you draw the line between the conceptual and the non-conceptual, meeting NC requires giving an account of some sort of basic cognition that cannot be reduced to mere dispositions but that, at the same time, can be accounted for in terms that do not presuppose the grasping of propositional fine-grained thoughts.

My proposal is to think of this more basic competence as a normative one and to model the minimal conceptual ability at issue as an ability to respond to standards of correct behavior in a way that suffices to distinguish between cases of absence of application and cases of misapplications of the standard^16^. The proposal is then to describe that behavior as a behavior of responding to specific standards of correction (hence being assessable as right or wrong according to those standards). Such an account must be one that conceives conceptual abilities in terms of more than mere causal mechanisms without thus committing to an explanatory gap concerning the emergence of propositional fine-grained articulated thought.

We can now define more precisely our question concerning the possibility of accounting for the normative constraint on conceptual abilities accommodating NC in the following terms: what features must a behavior have in order to count as a conduct that is sensitive to correctness patterns (unlike a behavior describable in merely dispositional terms) without thereby committing to it being explained as depending on propositionally articulated thought, thus leading to an evolutionary and explanatory gap.

Surprising as it might appear as first glance, I suggest that the crucial move to answer this question is to focus our attention into the kinds of interactions that basic intelligent creatures are able to deploy. This move is not completely novel in the literature. It was perhaps Dewey (1929) the first to emphasize that second-personal interaction is key to the learning of language- and this is a tradition that one can find exemplified in the later Wittgenstein as well as in Davidson’s and Brandom’s writings^17^. The crucial point to get clear about though is what *kind* of interaction we are referring to. In particular, we need to specify what features of the behavior at stake, if any (1) display sensitivity to standards of correction and (2) are both basic and at the same time sophisticated enough to meet NC.

A final further constraint on a proposal of this sort is for it to accommodate the available empirical evidence concerning language and concept acquisition. A first step could then be to take a look at the available evidence concerning language acquisition. The empirical study of the way in which such abilities are learned and deployed may help us identify the nature of the capacities involved. Furthermore, it is obvious from an empirical point of view – or at least denying it would be highly implausible – that small children do not have fine-grained articulated thought from the start, so the study of children’s development should exhibit the possibility of acquiring the capacity to grasp propositional articulated thoughts departing from previous non-propositional capacities that characterize the child’s earlier stages of development.

I propose that a natural candidate to account for the right kind of behavior capable of accommodating the normative constraint is what I call *sensitivity to correction*, that is the disposition to modify one’s behavior in the light of salient assessments of others with whom one is interacting. This claim still needs to gain support from empirical as well as conceptual grounds and I do try to provide such support in the remaining sections of this paper. Available evidence from developmental psychology will also provide some interesting cases of how this second-personal interaction can be conceived. Hence, while taking a look at empirical evidence, I expect to back up both my claim that a middle path between dispositionalism and interpretationism is in order and that such middle path is to be thought of in terms of a second-personal kind of interaction.

## EXAMINING THE EMPIRICAL EVIDENCE FROM DEVELOPMENTAL PSYCHOLOGY

As I said, one natural place to look for an answer to this question, framed with NC in mind, is the way children learn concepts.

Csibra and Gergely (2009) have argued that adults–children interaction is essential to the learning of conceptual content. They have conducted a number of experiments that suggests that there is a crucial difference in the subsequent behavior of the infants if they have learnt merely by observation – when the children are just observing the behavior of adults – or through being explicitly taught – i.e., when there is explicit demonstrative reference through the use of language to the objects the concepts apply to in a context in which the child is addressed. What they noted is that only in the latter case children generalize the result to all similar cases, while in the former they conceive of the case as contextually and situationally bound. This provides us a first indication that interaction plays a crucial role in learning and displaying conceptual abilities as opposed to other kind of learning, where no language is involved.

A second indication that the sort of interaction that humans are capable of might be key to the development of their conceptual abilities comes from primatology. Tomasello (1999) and Tennie et al. (2010) have claimed that chimpanzees are capable of emulating behavior but not of abstracting this conduct from the situational bound contexts in which they first perceive it. This means that while they are capable of imitating the use of tools in performing a specific task governed by their own interests and goals, they do not grasp the general meaning of the object nor of the end that is displayed in the behavior in a way that can be detached from the context and the objects they are observing and using in that specific occasion. This fits well with Csibra and Gergely’s (2009) studies suggesting that the interactive aspect of learning in humans involves a capacity to grasp the general, rule-like content of linguistic terms and behavior in a way that is not available to other creatures, and that this specific learning of general meanings takes place through particular training instances in the context of adult–child interactions, not being possible for children isolated from those interactions or for primates other than human who are not capable of those sorts of interactions (ibid)^18^.

Furthermore, Tomasello and Racokzy (2003) and Schmidt and Tomasello (2012) have studied the conduct of children regarding the enforcement of norms, and they observed that at two years of age children not only asses their behavior according to norms, accompanying what they do with statements of the sort “this is what we do” or “This is how it is done,” but also that they teach others (puppets but also adults that they identify as outsiders to the community) and that they complain when others do not conform to what they understand the social norm dictates in that particular situation. This means that children are ready to understand normative standards of behavior and to teach them to others at a very early stage of the development of their conceptual capacities and that they generalize the appropriateness of what they tend to do to all others with whom they are interacting, expecting them to act as they do and complaining if they refuse to do so.

How can this then help us to address NC, considering such behavior is exhibited by young children but not by other primates?

As I said before, there are a number of philosophical theories that have focused on the nature of human intersubjective exchanges to account for our capacity to grasp linguistic meanings. Haugeland (1990) and Brandom (1994) for example, have suggested that it is our attitude of treating a performance as right or wrong in particular contexts what makes that conduct right or wrong, and that this is a socially structured practice, in which we treat each other as committed and entitled or not to further actions as if we were playing a social game, the rules of which get specified by us treating the different moves as appropriate or not. Wittgenstein (1953) has also been read as defending a view according to which language should be thought of as a cluster of games that we play together and that it is internal to those games that certain moves are allowed or forbidden. The moves would then be correct or incorrect according to the game in the context of which they are assessed. Nevertheless, these theories are problematic if, as in Brandom’s theory, the moves of the game are thought to be propositionally articulated or if they imply interpretational stances on the part of the participants, as interpretationist accounts do. As I have argued before, such positions, if taken to be the whole story, turn out to be unable to meet NC. So I suggest that the right place to look at for is not the domain of interpretational theory but rather a different kind of interactionism, in particular interactionist phenomenologically based theories^19^.

Such theories start from one basic insight about the nature of social cognition: the fact that we are able to understand directly and correctly emotions on the face of others and their behavior as intentional and goal-oriented from the very first experiences of encountering others. This has been called “primary intersubjectivity.” It involves a kind of recognition of others that is displayed by newborns and that is characterized precisely by neither involving any kind of inferential cognitive mechanisms nor any mediation through articulated thoughts, such as attributing states to others. That notwithstanding, it involves more than just mere reactions to stimuli. More precisely, it involves grasping the meaning of the other person’s reactions. As Scheler famously described it: “that experiences occur there [in the other person] is given for us in expressive phenomena – […] not by inference, but directly, as a sort of primary “perception.” It is in the blush that we perceive shame, in the laughter joy” (Scheler, 1954, p. 10).

Phenomenology then provides us with a different route to understand the empirical findings of developmental psychology on the nature of normative behavior. It allows us to understand in what sense we are able to grasp the rightness or wrongness of what we are doing without committing us to think of this in a propositionally loaded way. According to these theories, based both in early development psychological studies and a phenomenologically based explanation of them, there is, from the very beginning of our lives, a way of tuning the other person’s emotions and it is that tuning, we might think, what first teaches us about the distinction between right and wrong, good or bad, this way or not-this-way.

Having taken a brief look at some recent works on Phenomenology and Developmental Psychology, we have found concurring support for the need to abandon the third-person perspective characteristic of interpretationism, but also the confinement within the first person perspective, characteristic of causalism. Such works suggest the convenience of prioritizing interlocutors’ interactions in face-to-face encounters in which the emotional recognition of the emotions of others might play a key role in our entry to language. It is in this domain, I argue, that we find the kind of behavior that allows distinguishing between conceptual mistakes and absence of application in a way that does not imply yet the reflective and explicit grasping of the standard to which we are nevertheless responding. In particular, I argue that it is our emotional response to approval and disapproval attitudes expressed in the interlocutors emotional behavior what allows us to learn from others language and criteria of correct use for words in contexts of use. Thus, this responsive behavior constitutes a kind of minimal conceptual competence vis-à-vis naturalist and normative constraints. How this allows us to accommodate the normative constraint answering at the same time to NC will be the topic of the next and final section.

## INTERACTION AND SENSITIVITY TO CORRECTION

As I have claimed, if the problems of interpretationism and causalism are taken seriously what we need to find is a form of behavior that is not reduced to causal reactions but does not presuppose the ability to entertain articulated thoughts. Furthermore, I have shown that taking into consideration the evidence from developmental psychology regarding the learning of language and norms, the right kind of behavior seems to be essentially interactive.

Advocators of the phenomenologically based interactionist theory usually draw a distinction between two different kinds of intersubjectivity that characterize capacities that are displayed at different stages in the child’s development. First, primary intersubjectivity (to be found from birth) is constituted by the ability to recognize emotions and reactions in other person’s faces without the use of any theoretical tool in face-to-face encounters. It is a capability that is primary, not acquired, but innate. The conduct of others is recognized as intentional, as directed toward an end. It involves temporal, auditive, and visual coordination with someone else with whom the baby is interacting. It is not substituted by other types of interaction but coexists with them, as a precondition for other abilities and as a complement of them. Later on^20^, children engage in secondary intersubjectivity, a kind of interaction that is characterized by the ability to identify objects and events in pragmatically meaningful contexts by shared attention mechanisms (based on the abilities gained through engaging in the previous kind of intersubjectivity). In this stage, children refer to the adults gaze when the meaning of an object is ambiguous or unclear. It is in the context of this kind of engagement with others that children learn a natural language by being taught and exposed to it in all sort of interactions^21^.

My suggestion is that the right place to look for the ability of self-correction is in the context of the capability of engaging in primary intersubjectivity^22^. It is in that domain that children display a disposition to respond to others, characterized by an attunement to their expectations and an ability to shape their behavior as a way of responding and satisfying the demands of others, paying special attention to the kind of response that their behavior elicits in the adult. This kind of exchanges is possible through common engagements in face-to-face encounters where the emotions of both are directly perceptible for each other. The common contexts in which those interactions take place include objects and their properties, which, as the interaction evolves and the answers become more stable, begin to be understood as independent standing qualities and objects. Throughout this process, joint attention mechanisms among other capacities come into stage and help to develop an early stage conceptual understanding and a primitive form of using concepts that will later became much more sophisticated, gaining independence from particular assessments and responses. Nevertheless, they will never lose their connection with actual uses and assessments of others.

How can we then distinguish between conceptual mistakes and absence of application in this early stage of development? In the previous section, I have examined some relevant work in developmental psychology on the nature of normative behavior and learning. Those studies suggest that interactions are key in that they elicit and display normatively informed behavior that is exhibited in the way in which children respond to adults in learning through two basic attitudes: *generalizing* (what they take to be *correct*) and *enforcing* on others the norm (actively correcting each other, showing that they are not only passively responding to the environment but spontaneously conceiving of what they are doing as an *standard of correction* to which themselves and all others are *supposed to conform*). Accordingly, in the context of the kind of interaction just described, I suggest there is a specific ability that constitutes a better candidate than mere reactions or articulated thought to meet NC. I call such ability *sensitivity to correction*. It can be defined as the disposition to modify one’s own behavior regarding the application of a specific concept in the light of the consent and dissent of others with whom one is interacting in face-to-face encounters. Sensitivity to correction so defined is precisely the feature of human behavior that allows us to accommodate the normativity constraint without abandoning the naturalistic conditions of adequacy that constitute NC.

When characterizing the different levels involved in self-correction (a pervasive feature of normative behavior), I mentioned: (a) the application of concepts (the actions of applying or misapplying a concept), (b) The ability to evaluate (a) and (c) the modification of (a) according to the results of (b). Both causalist and interpretationist account of conceptual capacities fail to provide a consistent answer to account for the difference between conceptual mistake and absence of application overemphasizing one of the elements, (a) as a model for (b) in the case of causalism, (b) as the all-encompassing interpreter’s perspective in the case of interpretationism. My proposal, on the contrary, is to think of level (b) as constituted by *sensitivity to correction*, that is the ability to correct and monitor our own action in the light of the reactions of others toward those very actions^23^. In this case (a) corresponds to a kind of behavior that displays intentionality, being directed toward an object to which the behavior is responding and (b) corresponds to the dimension in which we self-monitor our reaction to the object by tuning it to the way other reacts to us and our directed behavior. Sensitivity to correction is a social disposition, that is, a disposition to tune our behavior to the assessments and normative feedbacks we get from others in particular interactions. It is then an evaluative attitude that involves the perceiving and attunement to the approval or disapproval from others. Finally, corresponding to (c), the way in which we apply concepts is of course modified through the assessments involved in (b): actually, we may say, assessing our conduct amounts – at least in the most early stages of the acquisition of language and conceptual abilities – to modifying it according to the approval or disapproval of others.

We may now characterize the difference between conceptual mistakes and absence of application given the framework I have just presented. This distinction will take different shapes along the different stages involved in learning and grasping concepts. It will first consist in the ability to correct ourselves by tuning the other person’s assessments (monitoring myself through you, trying to make my own the perspective of the other with whom the interaction is taking place). It is a self-monitoring mechanism based upon the convergence of joint attention mechanisms that identify what is salient in the context and of the other’s monitoring of my own performance; the individual monitors her conduct taking into account both what she is directed to (level a) and assessing it in accordance to the assessment of others (level b), by then modifying the behavior accordingly (level c). It is precisely through responding to the other’s gaze and his attitudes of approval or disapproval that a criteria for the application of a concept in practice can be thought to be in place, as a standard of correction, hence distinguishing the case at stake from one in which the concept is not relevant at all, a case of absence of application. The concept in question would be poor in content at this point and its boundaries blurry. Thus conceptual competence at this stage is understood as a minimum conceptual understanding: but that minimum is exhibited precisely by the fact that the behavior is sensitive to a distinction between right and wrong ways of acting according to specific standards of correction (concepts), and this in turn is equivalent to there being a right way of acting in the world that the other and I share. Sensitivity to correction is, we may say, the phenomenological exhibition of the normativity of concepts. We can thus distinguish conceptual mistakes from cases of absence of application in that the subject is responding to the assessment of his behavior by modifying it accordingly as will not be the case if it were a case of absence of application. So, what makes the crucial difference is sensitivity to correction, a sensitivity that is displayed in actual interactions. Now, as learning progresses, self-correction gains independence from the presence of actual assessors. And then the subject self-corrects herself according to different actual or imagined scenarios and perspectives that she can reenact. Sociability is still a pervasive and crucial element of self-correcting behavior but is now exhibited as the very idea that I can be wrong according to different standards (which equates to the idea that there are other perspectives)^24^.

Finally, it is time to consider whether the tools just introduced are capable of properly meeting NC when accounting for the normative dimension involved in concept use. I cannot provide in this paper a detailed and all-encompassing answer to NC but, as it will be shown next, this proposal can give a proper general strategy to meet NC. This general strategy consists in identifying sensitivity to correction as the middle step between mere causal responses to the environment and contentful propositional attitudes. While the latter imply complete independence, flexibility, detachability, and general inferential articulation; the former, on the contrary, only amounts to nomological covariances between states and objects that may fail given an open number of contextual variations. The important point is that between these two ends of the invisible line of development and evolution there are as well different intermediate stages.

Following this strategy, we can then give a general outline of the evolutionary path from creatures without language or thought to creatures with both abilities. In a first very elemental level there may only be reactions to stimuli, being error just a failure in causal mechanisms. The true normative dimension emerges precisely when sensitivity to correction enters into stage, displaying the ability to interact with others (same species, interspecies) in a primary interaction sort of exchange. This hypothesis is supported from the fact, underlined by many evolutionary theories (Tomasello, 1999, 2014; Tomasello and Racokzy, 2003), that the main evolutionary step that distinguishes humans from other species is the ability to engage in social interactions of a highly sophisticated nature. Accordingly, in this stage subjects are capable of applying concepts independently of stimuli and are capable of applying the same concept to different objects and different concepts to the same object^25^, ultimately gaining the capacity to associate language items with meanings (norms of use of sounds and marks). Thus, the well-acknowledged idea of sociality as the trait characteristic of the emergence of the human^26^, when understood in terms of sensitivity to correction, can also explain the emergence of normative behaviors without any explanatory gap. The possibility of interpreting others and ourselves explicitly as following or failing to follow certain norms or rules, an ability that involves already propositionally articulated thoughts, is to be gained by engaging in earlier forms of sociality^27^.

A similar point can be made regarding the question of ontogenesis, where practical engagements with others in face-to-face encounters (primary intersubjectivity) that display a primitive form of sensitivity to correction progressively lead to secondary intersubjectivity, as a form of interaction involving shared attention mechanisms, monitoring and correcting, in the context of which language is learned. Learning is a process in which the child eventually gets to be a competent user. At the beginning she may need guidance and mainly self-correct when assessed negatively but later on, she will try herself to repeat this correcting behavior thus generalizing what is learnt and gaining autonomy in self-assessing her own behavior. Once again, the third-personal interpretative stance can only get into the picture much later once the full inferential capacity and the capability of complex interpretation processes are in place.

## CONCLUDING REMARKS

I have claimed that two of the most popular theories that account for conceptual competence fail when considered against the background of both the NC, i.e., the challenge of accounting for both the ontogeny and phylogeny of conceptual thought without explanatory or evolutionary gaps, and the normative constraint, i.e., the distinction between conduct that is guided by an standard of correction and the conduct that can only be externally assessed as responding to concepts.

Following some insights from developmental psychology and phenomenology, I have presented an alternative framework, interactionist theory, in the context of which the normativity constraint is accommodated in the domain of actual interactions with others in the learning of language and concepts. My central claim was that sensitivity to correction is a social, evaluative disposition that tunes us to other people’s assessments of our behavior in actual interactions and allows us to learn from them standards of correction for our actions. This kind of disposition is what makes the difference evolutionarily and in terms of individual development. The fact that human sociality is the main difference between us and other species is pervasively accepted and has independent grounds in evolutionary studies. If we can make sense of the connection between conceptual informed behavior and social behavior, as we have proposed we can, then this gives indirect support to the idea that this might be the crucial step in the evolutionary story of the human species. As for the case of human learning, I argued that recent studies in developmental psychology suggest that it is precisely our ways of engaging with others and understanding them what underlies our capacity to learn from each other the kind of general and abstract meanings that we then deploy in our social lives. The so often underlined social character of human life may find in the idea of sensitivity to correction a further specification capable of illuminating the way in which language and thought emerge.

## Conflict of Interest Statement

The author declares that the research was conducted in the absence of any commercial or financial relationships that could be construed as a potential conflict of interest.

## Footnotes

1 It was J. Levine the first to use the expression in the context of the discussion of reductivist accounts of the mind. He said: “In the end, we are right back where we started. The explanatory gap argument doesn’t demonstrate a gap in nature, but a gap in our understanding of nature. Of course a plausible explanation for there being a gap in our understanding of nature is that there is a genuine gap in nature. But so long as we have countervailing reasons for doubting the latter, we have to look elsewhere for an explanation of the former” (http://cognet.mit.edu/posters/TUCSON3/Levine.html). Even if Levine was referring to another aspect of the mind, the point still applies in relation to the development and evolution of conceptual capacities. The use of the expression “evolutionary gap” is meant to emphasize the need of having an explanation of how certain capacities evolved from others, instead of postulating a gap in nature. “Explanatory gap” refers to what Levine calls a gap in our understanding, i.e., the insufficiency of a certain set of explanatory tools to infer or otherwise explain conceptual capacities.

2 “Justification” in this condition is to be understood in broad terms. Thus, it is meant to cover a broad range of explanatory accounts of those attributions, not merely accounts that will take those attributions to be literally true. There is nevertheless a minimal constraint that justification places in these explanations. It requires that the explanation of the attributions is based on the abilities displayed in the behavior of the organism to which the attributions are made.

3 For a full list of necessary conditions for the possession of conceptual abilities, see Camp (2009) and Scotto (2010).

4 Of course there are cases like (ii) in which we say that John should have been adding and *ante* that there is then an *error of performance*. But in such cases, what we mean is that *he should have known the concept*: the problem resides precisely in the absence of application of that concept and not a misapplication of it.

5 Forbes (1984), Ginet (1992) and Fodor (1998) are some of the advocates of this approach, although it is much more broadly accepted.

6 According to the kind of concept, conditions C will vary. They may for instance include normality in the subject’s cognitive functions as well proper external conditions, so for example, were the concept a perceptual one, then proper conditions of illumination will be included as well as the proper functioning of the visual system.

7 Kripke (1982) and Wright (1989) have also argued for the same conclusion. The main claim, as we will see, is that the causalist way of specifying conceptual competence is circular, in as far as it presupposes the very concept that is supposed to be specifying by the identification of the relevant dispositions. For a discussion of this see Satne (2005, chapter 3).

8 One might think that I am presupposing that self-correction as I define it is a personal-level concept and thus unable to challenge subpersonal accounts of conceptual abilities. On the contrary, the definition is neutral with respect to this. I thank one of the anonymous referees for pressing this point.

9 Fodor (1990a) specifies the concept in question in terms of higher order relations of asymmetrical dependency between causal relations of this sort. But the problem reappears in a slightly different form: postulating asymmetrical relations between causal relations in the absence of a naturalistic explanation of why those relations should hold merely restates the problem at issue (Hutto, 1999, 2009, pp. 47–48, p. 22; Cummins, 1989).

10 Again, the account could sensically hold that this mechanism is to be understood as operative in a subpersonal level.

11 Another relevant candidate to account for the normativity of conceptual abilities is teleosemantics, a model that appeals to the notion of biological function and the evolutionary history of the organisms to explain representational content. I would not consider this proposal in detail in this paper. The main reason is that as Fodor (1990b) has argued, biological function is not sufficient for intensionality: we can explain the behavior at issue according to one concept or other as long as they are co-extensional in the relevant *de facto* situations. In the present context this would amount to a failure to distinguish between conceptual mistakes according to a concept and absence of application of that concept. For a detailed treatment of Teleosemantics and the problems it rises for explaining conceptual content see Hutto and Satne (2014), where I argue that a story of that sort is part of the explanation of the relevant capacities but not yet sufficient to account for the normativity of conceptual content.

12 Davidson (1975, 1982, 1984, 1986, 1992, 1994, 2001, 2005), Stalnaker (1984), Dennett (1991), and Brandom (1994) are some of the main advocates of this approach. Further specifications are required that distinguish their positions. I may dispense of introducing such distinctions here since nothing especially important for the arguments presented in this section follows from drawing these distinctions.

13 I will be following mainly Davidson’s presentation of the central traits of the theory although a similar case, with correspondent adjustments, can be made for Dennett’s, Stalnaker’s and Brandom’s accounts.

14 For a proposal exactly along these lines, see Hutto (2008).

15 For an overview of the main views that endorse non-conceptual content and discussions thereof, see York (2003).

16 Some may think that responding to a specific standard of correction should not be classified as a conceptual behavior, but instead representational, and that we should reserve the term “conceptual” for propositional articulated thought and behavior. At this point, this will perhaps be a terminological issue. For a proposal along those lines, see Schmitz (2012, 2013).

17 Wittgenstein (1953), Davidson (1984, 2001), and Brandom (1994). Also Hutto and Myin (2013).

18 Csibra and Gergely (2009) have called this specific aspect of the way human beings teach and learn from each other “natural pedagogy.” Tomasello (1999, 2014) argues that primates are incapable of engaging in joint action with other primates or humans because they lack the ability to form intentions about other individuals intentions. Here I am not committing to the particular explanation Csibra and Gergely (2009) give of the abilities in which this sort of interactions are based, nor to Tomasello’s explanation, in both cases highly sophisticated Theory of Mind abilities seem to be required. Regardless of their explanations, the evidence points toward a key role for interaction in the ability to learn and apply conceptual contents. With the idea of meeting NC, I provide a different and less demanding understanding of what is at issue in interaction that accounts for these differences.

19 Trevarthen (1978, 1979), Hobson (2002), Reddy (2008), and Rochat (2012) have defended and developed this theory from a psychological point of view. Gallagher (2001, 2004, 2007), Gallagher and Hutto (2008) and Gallagher and Zahavi (2008) have provided reasons in favor of if from the philosophical one.

20 There is some debate about when exactly this happens among advocates of the interactionist theory, ranging from 6 to 18 months of age depending on the author.

21 Gallagher and Hutto (2008) have claimed that narratives play a crucial role in the way in which children learn different perspectives and build a conception of themselves and of others that is enriched *vis-à-vis* the primary and emotional sort of engagement characteristic of the initial encounters with others. Even if this may be so, a previous question to be made, following our previous considerations, is how is it that children learn to respond to concepts as standards to assess their own conduct.

22 Varga and Gallagher (2012) have claimed that the notion of recognition, as an interpersonal demand, that occupies a central role in the discussions of moral normativity, should be traced back to its primary location in this first strongly psychologically based kind of interaction with others. I am claiming that this recognitional competence plays a role in conceptual normativity as well.

23 According to this view, what is directly perceived are emotions, associated with positive and negative reactions toward other’s behavior when conceiving it correct or incorrect. So by extension, understanding such assessment can be thought as based on the ability to perceive these positive and negative emotions and tune to them by changing one’s behavior accordingly. The intentional directed behavior of the adults or peers, that is also perceived, will also play a key role in understanding what kind of performance is expected. I am grateful to one of the anonymous reviewers for pressing this point.

24 It is important noticing that contrary to Hutto’s (1999) and Davidson’s (2001) view the idea is not that perceiving other perspectives as such gives a normative dimension to what I am doing, but that first I *attune* my behavior to what others *expect* from me and only latter the difference of perspectives can became salient and object of my own reflection. This last possibility is only present when there is also the capability of grasping explicitly the standards that this other perspectives represent and how they stand to the behavior being assessed.

25 This is the satisfaction of a simplified version of the Generality Constraint (see Camp, 2009). All these abilities together amount to the acquiring of minimal conceptual capacities (for conditions on minimal conceptuality, see Camp, 2009; Scotto, 2010).

26 See Sterelny (2012) and Tomasello (2014).

27 I am making a distinction between three paradigmatic and different abilities: (i) causal responses to the environment; (ii) sensitivity to correction in interaction; (iii) entertaining of propositionally articulated thoughts. This distinction is schematic and it is meant to distinguish important milestones in development and evolution. But this threefold classification should not be taken to characterize one stage in development as opposed to others. On the contrary, those abilities appear in Interactionist Theory only as paradigmatic of some stages that give rise to the others (and multiple other intermediate ones in between) by ways of progressive complexity. Accordingly, each stage in evolution and development integrates in different manners previous stages not by replacing them but by complementing them with new abilities.

## References

[B1] Boghossian P. (1989). The rule following considerations. *Mind* 98 507–549 10.1093/mind/XCVIII.392.507

[B2] Brandom R. (1994). *Making It Explicit*. Cambridge, MA:Harvard University Press

[B3] Camp E. (2009). Putting thought to work: concepts, systematicity and stimulus-independence. *Philos. Phenomenol. Res.* 78 275–311 10.1111/j.1933-1592.2009.00245.x

[B4] Cheney D. L. Sey-Farth R. M. (2007). *Baboon Metaphysics. The Evolution of a Social Mind.* Chicago:The Chicago University Press 10.7208/chicago/9780226102429.001.0001

[B5] Csibra G. Gergely G. (2009). Natural pedagogy. *Trends Cogn. Sci.* 13 148–153 10.1016/j.tics.2009.01.005 19285912

[B6] Cummins R. (1989). *Meaning and Mental Representation.* Cambridge, MA:MIT Press

[B7] Davidson D. (1975). “Thought and talk,” in *Inquiries into Truth and Interpretation.* Oxford: Oxford University Press 155–170

[B8] Davidson D. (1982). “Rational animals,” in *Subjective, Intersubjective, Objective.* Oxford: Oxford University Press 95–106

[B9] Davidson D. (1984). *Inquiries into Truth and Interpretation.* Oxford: Oxford University Press

[B10] Davidson D. (1986). “A nice derangements of epitaphs,” in *Truth, Language and History* Oxford: Clarendon Press 89–107

[B11] Davidson D. (1992). “The second person,” in *Subjective, Intersubjective, Objective.* Oxford: Oxford University Press 107–121

[B12] Davidson D. (1994). “The social aspect of language,” in *Truth, Language and History.* Oxford: Clarendon Press 109–125

[B13] Davidson D. (2001). *Subjective, Intersubjective, Objective*. Oxford:Oxford University Press 10.1093/0198237537.001.0001

[B14] Davidson D. (2005). *Truth, Language and History.* Oxford:Clarendon Press 10.1093/019823757X.001.0001

[B15] Dennett D. (1991). *Consciousness Explained.* London:Penguin

[B16] Dewey J. (1929). *Experience and Nature.* La Salle, IL:Open Court

[B17] Fodor J. (1990a). *A Theory of Content and Other Essays.* Cambridge, MA:MIT Press

[B18] Fodor J. (1990b). “Information and representation,” in *Information, Language and Cognition* ed. Hanson P. P. Vancouver:University of British Columbia Press

[B19] Fodor J. (1998). *Concepts: Where Cognitive Science Went Wrong*. New York:Oxford University Press 10.1093/0198236360.001.0001

[B20] Forbes G. (1984). Skepticism and semantics knowledge. *Proc. Aristot. Soc.* 1983–1984, 223–237

[B21] Gallagher S. (2001). The practice of mind: theory, simulation, or primary interaction. *J. Conscious. Stud.* 8 83–108

[B22] Gallagher S. (2004). Understanding interpersonal problems in autism: interaction theory as an alternative to theory of mind. *Philos. Psychiatr. Psychol.* 11 199–217 10.1353/ppp.2004.0063

[B23] Gallagher S. (2007). “Phenomenological and experimental research on embodied experience,” in *Body, Language and Mind* Vol. 1 eds Ziemke T. Zlatev J. Frank R. Dirven R. (Berlin:Mouton de Gruyter) 241–263

[B24] Gallagher S. Hutto D. (2008). “Understanding others through primary interaction and narrative practice,” in *The Shared Mind: Perspectives on Intersubjectivity* eds Zlatev J. Racine T. P. Sinha C. Itkonen E. (Amsterdam:John Benjamins) 17–38

[B25] Gallagher S. Zahavi D. (2008). Replies, in symposium on Gallagher and Zahavi, the phenomenological mind. *Abstracta* 2 86–107

[B26] Ginet C. (1992). The kripkenstein sceptical paradox and solution. *Midwest Stud. Philos.* 17 53–73 10.1111/j.1475-4975.1992.tb00142.x

[B27] Haugeland J. (1990). The intentionality all-stars. *Philos. Perspect.* 4 383–427 10.2307/2214199

[B28] Hobson P. (2002). *The Cradle of Thought.* London: Palgrave-Macmillan

[B29] Hutto D. (1999). *The Presence of Mind.* Philadelphia, PA: John Benjamins 10.1075/aicr.17

[B30] Hutto D. (2008). *Folk-Psychological Narratives. The Sociocultural Basis of Understanding Reasons.* Cambridge, MA: MIT Press

[B31] Hutto D. (2009). Mental representation and consciousness. *Encyclopedia Conscious.* 2 19–32 10.1016/B978-012373873-8.00050-5

[B32] Hutto D. Myin E. (2013). *Radicalizing Enactivism. Basic Minds Without Content.* Cambridge, MA: MIT Press

[B33] Hutto D. Satne G. (2014). The natural origins of content. *Philos. Q. Isr.* (in press)

[B34] Kripke S. (1982). *Wittgenstein on Rules and Private Language.* Cambridge, MA: Harvard University Press.

[B35] Reddy V. (2008). *How Infants Know Minds.* Cambridge, MA: Harvard University Press

[B36] Rochat P. (2012). “Early embodied subjectivity and inter-subjectivity,” in *Dimensoes da Intersubjectividade* eds Coelho N. E. Jr. Salem P. Klautau P. (Sao Paolo: Escuta) 280

[B37] Satne G. (2005). *El Argumento Escéptico. De Wittgenstein a Kripke*. Buenos Aires: Grama Ediciones

[B38] Scheler M. (1954). *The Nature of Sympathy.* London: Routledge & Kegan Paul

[B39] Schmitz M. (2012). “The background as intentional, conscious, and nonconceptual,” in *Knowing Without Thinking: Mind, Action, Cognition and the Phenomenon of the Background* ed. Radman Z. (Palgrave: Macmillan) 57–82

[B40] Schmitz M. (2013). “Social rules and social background,” in *The Background of Social Reality: Selected Contributions from the Inaugural Meeting of ENSO. Studies in the Philosophy of Sociality* eds Schmitz M. Kobow B. Schmid H.-B. (Berlin: Springer) 107–126 10.1007/978-94-007-5600-7_7

[B41] Schmidt M. Tomasello M. (2012). Young children enforce social norms. *Curr. Dir. Psychol. Sci.* 21 232–236 10.1177/0963721412448659

[B42] Scotto C. (2010). Basic concepts. *Paper Presented at the Workshop on Concepts and Perception*, Córdoba, Argentina

[B43] Stalnaker R. (1984). *Inquiry.* Cambridge, MA: MIT Press

[B44] Sterelny K. (2012). *The Evolved Apprentice. How Evolution made Humans Unique.* Cambridge, MA: MIT Press

[B45] Tennie C. Call J. Tomasello M. (2010). Evidence for emulation in chimpanzees in social settings using the floating peanut task. *PLoS ONE* 5:e10544 10.1371/journal.pone.0010544 PMC2868881 20485684

[B46] Tomasello M. (1999). *The Evolution of Human Cognition.* Cambridge, MA: Harvard University Press

[B47] Tomasello M. (2014). *The Natural History of Human Thinking.* Cambridge, MA: Harvard University Press

[B48] Tomasello M. Racokzy H. (2003). What makes human cognition unique? From individual to shared to collective intentionality. *Mind Lang.* 18 121–147 10.1111/1468-0017.00217

[B49] Trevarthen C. (1978). “Secondary intersubjectivity: confidence, confiding and acts of meaning in the first year,” in *Action Gesture and Symbol, The Emergence of Language* ed. Lock A. (London: Academic Press) 588

[B50] Trevarthen C. (1979). “Communication and cooperation in early infancy: a description of primary intersubjectivity,” in *Before Speech: The Beginning of Interpersonal Communication* ed. Bullowa M. (Cambridge: Camrbidge University Press) 410

[B51] Varga S. Gallagher S. (2012). Critical social philosophy, Honneth and the role of primary intersubjectivity. *Eur. J. Soc. Theory* 15 243–260 10.1177/1368431011423606

[B52] Wittgenstein L. (1953). *Philosophical Investigations* eds Anscombe G. E. M. Rhees E. trans. Anscombe G. E. M. (Oxford:Blackwell).

[B53] Wright C. (1989). “Wittgenstein’s rule-following considerations and the central project of theoretical linguistics,” in *Rails to Infinity* ed. Wright C. (Cambridge, MA: Harvard University Press) (Reprinted by Wright in 2001)

[B54] York G. (ed.) (2003). *Essays on Non-Conceptual Content.* Cambridge, MA:MIT Press

